# Comparison of the perioperative time courses of matrix metalloproteinase-9 (MMP-9) and its inhibitor (TIMP-1) during carotid artery stenting (CAS) and carotid endarterectomy (CEA)

**DOI:** 10.1186/s12883-018-1133-1

**Published:** 2018-08-29

**Authors:** Ákos Mérei, Bálint Nagy, Gábor Woth, János Lantos, Ferenc Kövér, Lajos Bogár, Diána Mühl

**Affiliations:** 1 Department of Anaesthesiology and Intensive Therapy, Medical School, University of Pécs, Ifjúság Str. 13, Pécs, HU-7624 Hungary; 2 Department of Surgical Research and Techniques, Medical School, University of Pécs, Szigeti Str. 12, Pécs, HU-7624 Hungary; 3 Medical Skills Lab, Medical School, University of Pécs, Szigeti Str. 12, Pécs, HU-7624 Hungary; 4 Department of Operational Medicine, Medical School, University of Pécs, Szigeti Str. 12, Pécs, HU-7624 Hungary; 5 Department of Neurosurgery, Medical School, University of Pécs, Rét Str. 2, Pécs, HU-7623 Hungary

**Keywords:** Carotid stenting, Carotid endarterectomy, Matrix metalloproteinase, MMP-9, TIMP-1

## Abstract

**Background:**

Our aim was to compare the perioperative time courses of matrix metalloproteinase-9 (MMP-9) and its inhibitor (TIMP-1) in during carotid endarterectomy (CEA) and carotid artery stenting (CAS).

**Methods:**

In our prospective study, twenty-five patients who were scheduled to undergo CAS were enrolled. We used a matched, historical CEA group as controls. Blood samples were collected at four time points: T1: preoperative; T2: 60 min after stent insertion; T3: first postoperative morning; and T4: third postoperative morning. Plasma MMP-9 and TIMP-1 levels were measured by ELISA.

**Results:**

In the CEA group, the plasma levels of MMP-9 were significantly elevated at T3 compared to T1. In the CAS group, there was no significant difference in MMP-9 levels in the perioperative period. MMP-9 levels were significantly higher in the T3 samples of the CEA group compared to the CAS group. Significantly lower TIMP-1 levels were measured in both groups at T2 than at T1 in both groups. MMP-9/TIMP-1 at T3 was significantly higher than that at T1 in the CEA group compared to both T1 and the CAS group.

**Conclusions:**

CAS triggers smaller changes in the MMP-9-TIMP-1 system during the perioperative period, which may correlate with a lower incidence of central nervous system complications. Additional studies as well as cognitive and functional surveys are warranted to determine the clinical relevance of our findings.

**Trial registration:**

NIH U.S. National Library of Medicine, Clinicaltrials.gov,NCT03410576, 24.01.2018, Retrospectively registered

## Background

Ischaemic stroke is one of the leading causes of death, dementia and disability in the developed world [1, 2]. Among stroke survivors, the risk of recurrent attacks remains high. Significant stenosis of the internal carotid artery is a well-known risk factor of ischaemic stroke [3]. Intervention of the stenotic carotid artery can decrease the risk of stroke. The gold standard intervention is carotid endarterectomy (CEA), but carotid artery stenting (CAS) is a less invasive therapeutic method with increasing popularity [4–6]. Although both methods have well-known complications, clinical trials have found that the risk of death or recurrent stroke within 30 days of surgery in symptomatic patients was higher among the CAS patients than among the CEA patients [7–12]. According to the 2014 AHA/ASA stroke prevention guideline for low- or average-risk symptomatic patients, CAS is an alternative to CEA when the internal carotid artery stenosis is greater than 70% by non-invasive imaging or greater than 50% by catheter-based imaging and if the anticipated rate of periprocedural stroke or death is less than 6% [13]. For patients younger than 70 years old, the risk of periprocedural complications (stroke, myocardial infarction or death) and the long-term risk of ipsilateral stroke is equal whether they undergo CAS or CEA [13]. CAS is also a reasonable treatment modality among patients with symptomatic severe stenosis (> 70%) and concurrent anatomic or medical conditions that increase the risks of surgery or in cases with specific circumstances such as radiation-induced stenosis or restenosis after CEA [13].

The gold standards of periprocedural stroke diagnosis are clinical parameters and neuroimaging. In recent years, various biomarkers of neural damage have been tested and validated; however, a single biomarker that is capable of identifying neural damage is not yet available [14]. Matrix metalloproteinases (MMPs) and their tissue inhibitors (TIMPs) have already been examined in CEA- and CEA-associated periprocedural stroke [15, 16]. MMPs play a crucial role in extracellular matrix turnover, degradation and remodelling. In addition, the effects of MMPs in signalling pathways have been confirmed and evaluated in various physiological and pathophysiological conditions [17–19]. Matrix metalloproteinase-9 (MMP-9) is a gelatinase enzyme and a marker of inflammation. Although it lacks specificity in the monitoring of neuronal damage, increased perivascular tissue levels and microglial production of MMP-9 were observed in acute stroke patients. Higher MMP-9 levels have been found to be associated with increased oxidative stress, apoptosis, blood-brain-barrier (BBB) dysfunction and development of cerebral oedema [20]. According to in vitro results, TIMP-1 can play a protective role in neuronal apoptosis and neurotrophic action, but in vivo data are controversial, as the possibility of secondary TIMP-1 increases as a result of BBB injury [21]. The interaction between MMPs and TIMPs is stoichiometric, and therefore, MMP/TIMP ratios can be used to reflect MMP-TIMP activity.

In one of our earlier studies, we examined the time course of CEA-related changes in MMP-9 and TIMP-1 [22]. Although Giuliani et al. investigated the levels of MMP-9 (and other biomarkers) in CEA patients versus CAS patients, the perioperative changes in the plasma levels of MMP-9 and TIMP-1 have not yet been evaluated [23]. The aim of our study was to compare the perioperative changes of the plasma levels of MMP-9 and TIMP-1 in CEA and CAS.

## Methods

Our study was carried out in accordance with the ethical guidelines of the 2008 Declaration of Helsinki at the Clinical Centre of University of Pécs in Hungary between October 2013 and November 2015. The study protocol was approved by the Institutional Scientific and Human Research Ethics Committee of the University of Pécs in Hungary. Following verbal and written information about the study, all enrolled patients provide their written informed consent to participate in our study. In total, twenty-five elective CAS patients were enrolled. The exclusion criteria were a diagnosis of malignant diseases, inflammatory and systemic autoimmune disorders, psychiatric disorders and previous debilitating stroke. For all patients, a preoperative anaesthetic assessment was routinely performed prior to the elective procedures. During this assessment, signs of ongoing infections or trauma were excluded.

In a study recently published by our workgroup, a group of 54 patients undergoing CEA was evaluated [22]. In the present study, a matched subgroup of 30 patients from the previously obtained data served as a historical control.

### Surgical procedures

A detailed description of the CEA procedure and blood sampling schedule is available in our prior publication [22]. The CEA group samples were collected at the following four time points (T1–4): T1, at the time of the insertion of the arterial line; T2, 60 min after cross-clamp release; T3, the first postoperative morning; and T4, the third postoperative morning.

### CAS operation

CAS was performed under regional anaesthesia with lidocaine. Pre- or intraoperative sedation was not performed on our CAS patients. After catheterising the right femoral artery, diagnostic angiography was performed in all cases. After the precise localisation of the stenosis, a guide catheter was inserted with the help of a hydrophilic guide wire. Then, through this guide catheter, a microwire was inserted through the stenotic area. Once the appropriate position was achieved, the lumen was opened with the gradual dilatation of the stent. During the dilatation, 0.5 mg of intravenous atropine was administered to prevent bradycardia. CAS patients were admitted to the neurosurgery ward after the procedure for postoperative monitoring. Blood samples from CAS patients were collected via an arterial cannula. Sampling was performed at three time points (T1–3): T1, at the time of the insertion of the arterial line; T2, 60 min after stent insertion; and T3: the first postoperative morning. The fourth sampling (on the third postoperative morning) was not performed because the patients were discharged from the hospital on the second postoperative day.

Plasma was isolated from heparin-anticoagulated blood samples by low speed centrifugation at 4 °C and stored at − 80 °C until they were analysed in a single batch at the end of the study. MMP-9 and TIMP-1 levels were measured with quantitative sandwich enzyme-linked immunosorbent assay (ELISA) techniques according to the manufacturer’s instructions (R&D Systems Inc., Minneapolis, MN, USA). Then, spectrophotometric (Multiskan Ascent microplate photometer, Type: 354, Thermo Electron Corporation, Waltham, MA, USA) reading of the absorbance at 450 nm was compared to standard curves. Plasma concentrations of MMP-9 and TIMP-1 were expressed as ng/ml.

### Statistical analysis

Non-parametric tests were used since the data distribution was found to be not normal by the Kolmogorov-Smirnov and Shapiro-Wilk tests. CEA patients and CAS patients were compared with the Mann-Whitney U test. Kruskal-Wallis one-way ANOVA with post hoc Dunn test was used to compare data of the different time points in both patient groups. The analyses were conducted by the Statistical Package for the Social Sciences (SPSS) Statistics software, version 21.0 (IBM Corporation, USA). Values of *p* < 0.05 were considered statistically significant.

## Results

Table 1 shows the demographic data, comorbidities, major complications and pre-existing medical conditions and treatments of the two analysed groups. There was no significant difference between the CAS group and the control CEA group in the number of patients enrolled, age, gender, medications, comorbidities and major complications. Age, gender, procedure laterality, previous stroke, presence of contralateral stenosis, prior ipsi- or contralateral surgery, smoking, pre-existing hypertension and diabetes treated with oral antidiabetic medication did not influence the plasma levels of MMP-9 and TIMP-1 at any time points. The plasma levels of MMP-9 among diabetic patients treated with insulin analogues were significantly higher in the T2 samples (Table 2). Lipid lowering agents and aspirin did not influence the plasma levels of MMP-9 and TIMP-1 at any time point. Baseline (T1) plasma MMP-9 levels of the patients treated with adenosine diphosphate (ADP) receptor antagonists were significantly lower (Table 3). Intraoperative hypo- or hypertension had no effect on plasma MMP-9 or TIMP-1 levels in the present study.

**Table 1:** Demographic and characteristic data of patients

Parameters	CEA	CAS
Patient number	30	25
Age (year)	65 ± 8	60 ± 7
Gender (male/female)	20/10	16/9
Laterality (right/left)	15/15	13/12
Degree of stenosis	83 ± 7.8%	79 ± 7.5%
Contralateral stenosis > 50% n (%)	8 (26.7)	9 (36)
Symptomatic ICA stenosis n (%)	16 (53.3)	17 (68)
Previous stroke n (%)	6 (20)	5 (20)
Time since stroke (months)	28 ± 10	27 ± 18
Previous transient ischaemic attack n (%)	10 (33.3)	12 (48)
Time since transient ischaemic attack (months)	6 ± 4	6 ± 3
Previous ipsilateral ICA procedure n (%)	4 (13.3)	7 (28)
Coexisting diseases		
Hypertension n (%)	29 (96.7)	23 (92)
Non-insulin-dependent diabetes mellitus n (%)	9 (30)	3 (12)
Insulin-dependent diabetes mellitus n (%)	3 (10)	2 (8)
Hyperlipidaemia n (%)	20 (66.7)	14 (56)
History of smoking n (%)	10 (33.3)	8 (32)
Medications		
Acetyl salicylic acid n (%)	16 (53.3)	20 (80)
ADP receptor antagonists n (%)	13 (43.3)	8 (32)
Lipid lowering agents n (%)	20 (66.7)	15 (60)
Intraoperative complications		
Hypertension n (%)	9 (30)	1 (4)
Hypotension n (%)	8 (26.7)	1 (4)
Transient shunt n (%)	3 (10)	0 (0)
Bleeding (> 100 ml) n (%)	2 (6.7)	0 (0)
Transient ischaemia n (%)	4 (13.3)	0 (0)
Stroke n (%)	0 (0)	0 (0)
Postoperative complications		
Bleeding (> 100 ml) n (%)	2 (6.7)	1 (4)
Transient ischaemia n (%)	2 (6.7)	0 (0)
Stroke n (%)	0 (0)	0 (0)

Data are presented as the mean ± standard error of mean

*ICA* internal carotid artery, *ADP* adenosine diphosphate

**Table 2:** Effect of insulin analogue on plasma MMP-9 levels

		T1	T2	T3	T4
MMP-9 (ng/ml)	IA	327.5±33.5	585.3±170.6*	414.6±138.9	509.4±202.1
	Non-IA	273.7±26.8	238.3±31.8	359.0±42.9	336.2± 54.3

Data are presented as the mean ± standard error of mean

*IA* patients who were treated with insulin analogue, *non-IA* patients who were not treated with insulin analogue, *T1* preoperative values, *T2* 60 min after cross-clamp release/stent insertion, *T3* postoperative day 1, *T4* postoperative day 3; and

*p < 0.05 compared to non-IA patients

**Table 3:** Effect of ADP receptor antagonists on plasma MMP-9 levels

		T1	T2	T3	T4
MMP-9 (ng/ml)	ADP antag.	233.1 ± 20.7*	299.4 ± 66.6	382.0 ± 70.2	456.6 ± 101.4
	Non-ADP antag.	346.9 ± 49.3	246.7 ± 37.4	380.7 ± 53.0	320.6 ± 66.7

Data are presented as the mean ± standard error of mean

*ADP antag* patients who were treated with ADP receptor antagonists, *non-ADP antag* patients who were not treated with ADP receptor antagonists, *T1* preoperative values, *T2* 60 min after cross-clamp release/stent insertion, *T3* postoperative day 1, *T4* postoperative day 3; and

* *p* < 0.05 compared to non-ADP antag

**Fig. 1:**
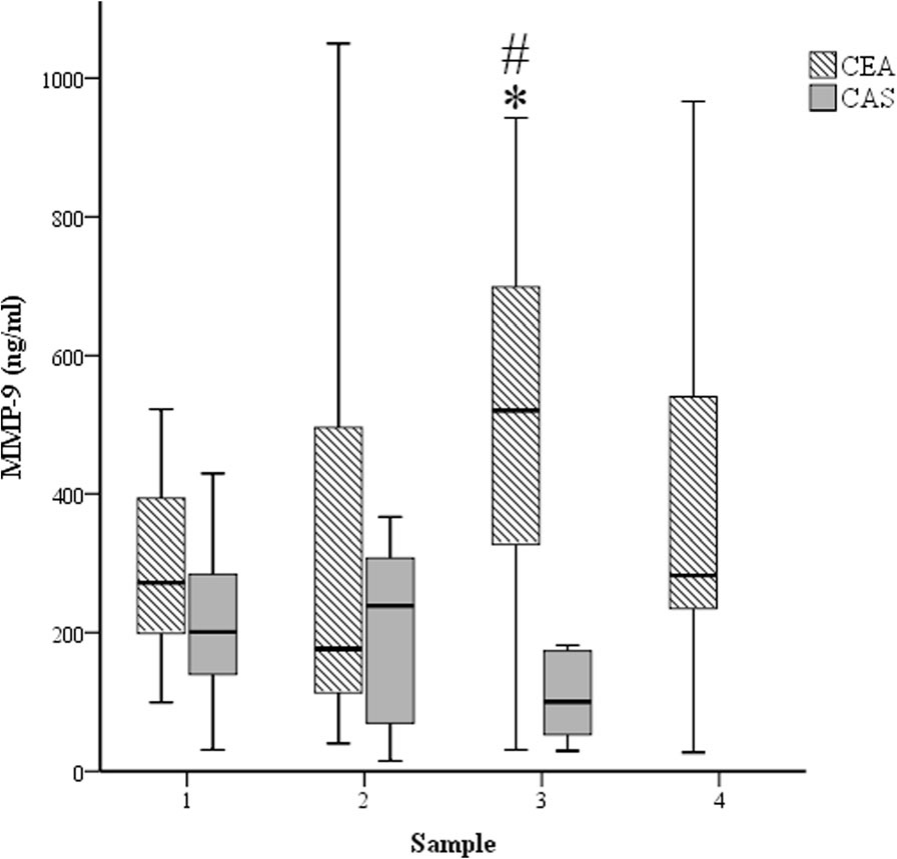
Changes in plasma MMP-9 concentrations in the two patient groups. CEA: carotid endarterectomy group (striated); and CAS: carotid artery stenting group (grey). Samples: 1, preoperative values; 2, 60 min after cross-clamp release/stent insertion; 3, postoperative day 1; and 4, postoperative day 3. *: *p* < 0.05 compared to sample 1; and #: *p* < 0.05 compared to CAS

In the CEA group, significantly higher plasma MMP-9 levels were measured at T3 compared to baseline (T1). There were no differences in the plasma MMP-9 levels in the CAS group at any time point (*p* > 0.05). In the T3 samples, plasma MMP-9 levels were significantly higher in the CEA group compared to the CAS group (Table 4 and Fig. 1).

**Table 4:** Plasma MMP-9, TIMP-1 and MMP-9/TIMP-1 levels in the study groups

	Group	T1	T2	T3	T4
MMP-9 (ng/ml)	CEA	290.9 ± 112.1	284.7 ± 247.5	488.6 ± 249.8*#	382.9 ± 285.4
	CAS	259.8 ± 244.9	239.1 ± 221.3	180.9 ± 159.7	–
TIMP-1 (ng/ml)	CEA	117.3 ± 43.2	81.7 ± 73.9*	88.5 ± 41.6	117.2 ± 52.3
	CAS	93.5 ± 30.9	61.7 ± 28.8*	70.7 ± 30.2	–
MMP-9/TIMP1	CEA	2.73 ± 1.37	4.39 ± 2.69	6.41 ± 3.86*#	3.40 ± 2.50
	CAS	2.78 ± 1.88	4.33 ± 3.05	2.26 ± 1.79	–

Data are presented as the mean ± standard error of mean

*CEA* carotid endarterectomy control group, *CAS* carotid angioplasty and stenting group, *T1* preoperative values, *T2* 60 min after cross-clamp release/stent insertion, *T3* postoperative day 1, *T4* postoperative day 3

* *p* < 0.05 compared to T1

# *p* < 0.05 compared to CAS

**Fig. 2:**
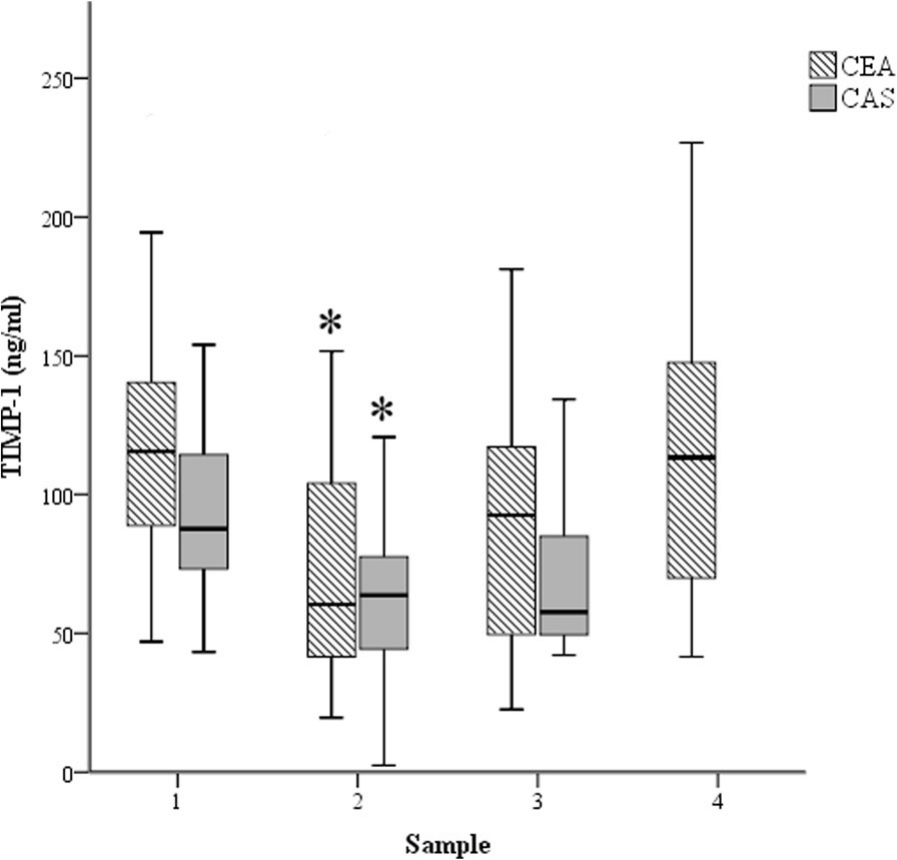
Changes in plasma TIMP-1 concentrations in the two patient groups. CEA: carotid endarterectomy control group (striated); and CAS: carotid artery stenting group (grey). Samples: 1, preoperative values; 2, 60 min after cross-clamp release/stent insertion; 3, postoperative day 1; and 4, postoperative day 3. *: *p* < 0.05 compared to sample 1

Significantly lower plasma TIMP-1 levels were measured in both groups at T2 compared to baseline (Table 4 and Fig. 2).

**Fig. 3:**
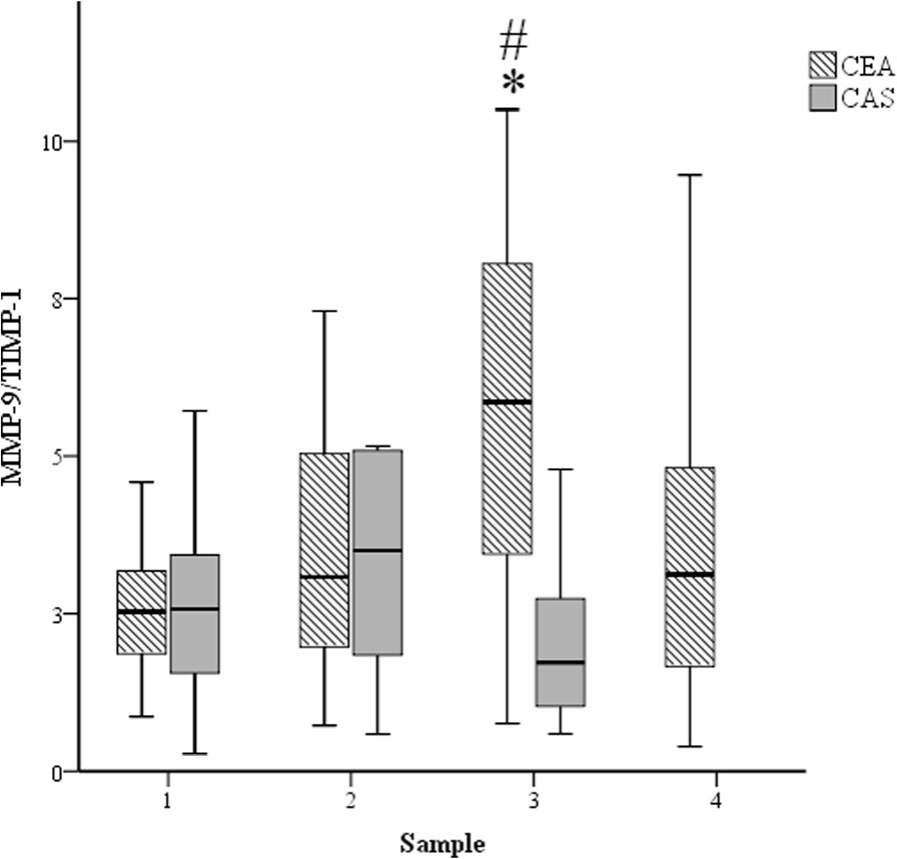
Changes in plasma MMP-9/TIMP-1 ratios in the two patient groups. CEA: carotid endarterectomy control group (striated); and CAS: carotid artery stenting group (grey). Samples: 1, preoperative values; 2, 60 min after cross-clamp release/stent insertion; 3, postoperative day 1; and 4, postoperative day 3. *: *p* < 0.05 compared to sample 1; and #: *p* < 0.05 compared to CAS

MMP-9/TIMP-1 ratios at T3 were significantly higher than baseline in the CEA group and the CAS group (*p* < 0.05) (Table 4 and Fig. 3).

## Discussion

Interventions on the extracranial carotid artery are relatively common vascular procedures worldwide. The gold standard intervention is still CEA; however, according to the 2014 AHA/ASA stroke prevention guideline, CAS can be equally effective and less invasive in selected cases [13]. Careful preoperative assessment and management are essential to identify the patients who may benefit from the less invasive nature of an endovascular operation compared to an open surgery.

Most studies on the MMP-9-TIMP-1 system in CEA and CAS have assessed changes at only a single time point [16, 23, 24]. Thus, our primary aim was to describe the time course of changes in MMP-9 and TIMP-1 levels during the perioperative period of CAS, compare the results to CEA and identify factors influencing these changes.

Muzahir et al. provided information regarding the correlation of MMP/TIMP levels with age, gender and diurnal activity [25]. There were no significant differences in the demographic data of the patients between the study groups, and all surgeries started in the morning; therefore, we could not ascribe the identified differences to population bias. Although hypo- and hypertension were more prevalent in the CEA group, these complications were promptly addressed.

The role of MMP-9 and TIMP-1 in cardiovascular remodelling is being intensively investigated [26, 27]. In paediatric hypertension, Niemirska et al. found sex-related elevations in the plasma concentrations of MMP-9 and TIMP-1 [28]. In our recent study, age, gender and pre-existing hypertension did not influence the plasma levels of MMP-9 and TIMP-1 at any time point.

The plasma level of MMP-9 has been found to be an independent risk factor for atherothrombotic events [29]. Among patients with coronary artery disease, concurrent type 2 diabetes mellitus is associated with higher plasma levels of MMP-9 [30]. This highlights the role of MMP-9 in diabetes-associated atherothrombosis. In our study, diabetes treated with oral antidiabetic medication did not influence the plasma levels of MMP-9 and TIMP-1 at any time point. Noticeably, the plasma levels of MMP-9 among diabetic patients treated with insulin analogues were significantly higher 60 min after reperfusion, but the difference was no longer detectable in the T3 or T4 samples. In contrast with our results, in vitro and animal studies have found that insulin treatment specifically inhibits MMP-9 expression [31]. Additional investigation of increased case numbers is necessary to resolve this contradistinction.

The mechanism by which ADP receptor antagonists influence plasma MMP-9 levels is not clear; however, the adenosine triphosphate (ATP) induced expression of MMP-9 has been investigated in different cell types [32, 33]. Choi et al. found that ATP induced microglial activation through the non-transcriptional activation of MMP-9, which can be inhibited by a P2Y receptor antagonist (including clopidogrel) in cell culture [34]. Sternberg et al. observed a reduction in MMP-9 plasma levels after clopidogrel administration in acute ischaemic stroke [35]. TIMPs can inhibit MMP-dependent platelet adhesion and aggregation. The effect of TIMPs on platelet aggregation can be enhanced by aspirin and ADP receptor antagonists. Reduced platelet aggregation caused by ADP receptor antagonist treatment may retroactively influence the expression of TIMPs [36].

After a transient ischaemic attack or ischaemic stroke, both the increase of MMP and decrease of TIMP levels can occur as a result of ischaemic damage and, later, as a result central nervous system repair [37, 38]. The significant increase of MMP-9 levels in the CEA group on the first postoperative day may indicate subclinical BBB dysfunction and/or microembolisation [16]. Using diffusion-weighted magnetic resonance imaging, Tedesco et al. observed a higher incidence of postprocedural microembolisation after CAS than after CEA [39], but Montorsi et al. found that the risk of microembolisation could be decreased with a proximal endovascular occlusion approach [40]. Another cause of the higher MMP-9 in the CEA group on the first postoperative day could be the longer carotid flow restriction compared to the endovascular procedure (CAS). The significant decrease of TIMP-1 in both groups during the study may be an indicator of increased extracellular matrix turnover following reperfusion [37, 38]. The higher MMP-9/TIMP-1 ratio on the first postoperative day in the CEA group originated from the higher plasma MMP-9 levels but seems to have been not significantly influenced by the TIMP-1 levels.

Limitations of our study: The extent of the resulting tissue damage from the two surgical procedures (CEA and CAS) are different. MMP-9 and TIMP-1 are commonly viewed as biomarkers of different pathophysiological conditions, but they also have biological properties and thus could potentially affect outcomes. As previously described by Agren et al., elevated MMP-9 levels are present in healing wounds, which may contribute to the elevated MMP-9 levels on the day after CEA surgery [41]. Another limitation of our study is that preoperative MRI was not routinely performed to determine the size of the previous ischaemic lesion; however, previous debilitating stroke was one of our exclusion criteria. Although the presence of contralateral carotid artery stenosis did not influence the plasma levels of MMP-9 and TIMP-1 at any time point, an examination of the effects of atherosclerosis in other arteries was not part of our study.

## Conclusions

According to our study, there were no significant differences in the incidence of neurological complications between the two groups during the perioperative period. However, the endovascular procedure triggers smaller changes in the MMP-9-TIMP-1 system, which may suggest a lower incidence of central nervous system damage. This finding may originate from a lower incidence of BBB dysfunction and/or microembolisation and a shorter carotid flow restriction time; however, the exact mechanism is not completely clear. Postoperative neuroimaging (such as MRI) may help identify subclinical changes; however, postoperative neuroimaging in a complication-free group was not performed. Additional studies in a larger group of patients, including a cognitive and functional survey at 1 and 6 months, are necessary to identify subtle differences in the functional outcome between the two procedures.

## Abbreviations

ADP: Adenosine diphosphate
ATP: Adenosine triphosphate
BBB: Blood-brain-barrier
CAS: Carotid artery stenting
CEA: Carotid endarterectomy
ELISA: Enzyme-linked immunosorbent assay
IA: Insulin analogue
ICA: Internal carotid artery
MMP-9: Matrix metalloproteinase-9
MMPs: Matrix metalloproteinases
TIMP-1: Tissue inhibitor of matrix metalloproteinase-1
TIMPs: Tissue inhibitor of matrix metalloproteinases
